# New framework to assess tracing and testing based on South Korea’s response to COVID-19

**DOI:** 10.1186/s12879-024-09363-4

**Published:** 2024-05-03

**Authors:** Junhyoung Kim, Suyoung Jo, Sung-il Cho

**Affiliations:** Seoul National University, Seoul, Korea

**Keywords:** COVID-19, Contact tracing, Testing

## Abstract

South Korea’s remarkable success in controlling the spread of COVID-19 during the pre-Omicron period was based on extensive contact tracing and large-scale testing. Here we suggest a general criterion for tracing and testing based on South Korea’s experience, and propose a new framework to assess tracing and testing. We reviewed papers on South Korea’s response to COVID-19 to capture its concept of tracing and testing. South Korea expanded its testing capabilities to enable group tracing combined with preemptive testing, and to conduct open testing. According to our proposed model, COVID-19 cases are classified into 4 types: confirmed in quarantine, source known, source unknown, and unidentified. The proportion of the first two case types among confirmed cases is defined as “traced proportion”, and used as the indicator of tracing and testing effectiveness. In conclusion, South Korea successfully suppressed COVID-19 transmission by maintaining a high traced proportion (> 60%) using group tracing in conjunction with preemptive testing as a complementary strategy to traditional contact tracing.

**Supplementary Information:**

The online version contains supplementary material available at 10.1186/s12879-024-09363-4.

## Background

Containment, suppression, and mitigation were proposed as coronavirus disease 2019 (COVID-19) pandemic response strategies [1]. Containment aims to eliminate community transmission (zero incidence for certain period of time beyond the latent period) via stringent interventions such as lockdown, border closure, and extensive tracing and large-scale testing [1]. Because containment is difficult to sustain in the long term, many countries implemented a suppression or mitigation strategy. Mitigation aims to minimize damage to high-risk populations and so allows a time-varying reproduction number (R_t_) > 1. By contrast, suppression aims to reduce R_t_ below 1 to minimize transmission [1, 2]. R_t_ is the average number of secondary cases of an infector during his or her infectious period and can be controlled by countermeasures and behavioral changes [3]. Lockdown, social distancing, mandatory mask wearing, restrictions on flights from high-risk countries, and temporary border closure were implemented in the United States, Argentina, and Uganda [1]. In addition, contact tracing and testing were proposed as essential case-based interventions in suppression strategy, but the approaches used differed according to national capabilities [1]. South Korea implemented a suppression strategy, which did not encompass lockdown or border closure.

South Korea’s COVID-19 control was remarkable compared to other countries that adopted a suppression strategy. In 2020, South Korea exhibited significantly lower daily new cases per million compared to the United States and Argentina, with maximum figures of 139, 4,489, and 2,109 cases per million, respectively. Additionally, the average daily new cases per million in South Korea were also notably lower than those observed in the United States and Argentina. South Korea minimized the number of confirmed cases by its suppression strategy through extensive contact tracing and large-scale testing (the 3Ts; tracing, testing, treatment) [4, 5]. South Korea achieved noteworthy outcomes, despite not implementing a rigorous containment strategy [4, 5].

Since the effectiveness of contact tracing is determined by the basic reproduction number (R_0_) and fraction of asymptomatic infection, contact tracing alone cannot counter COVID-19, which has high overall and silent transmission rates [6]. As a complementary measure, appropriate testing may be important. However, combined tracing and testing strategies have not been formulated [1, 2, 7], so a framework to maximize the effect of tracing and testing is needed. South Korea can serve as a reference for such a suppression strategy.

We performed a review of typical examples of South Korea’s response to COVID-19. In addition, we developed a conceptual model to explore effective tracing and testing strategies. The objectives of this study were to suggest general criteria for tracing and testing based on South Korea’s experience, and to propose a framework to assess tracing and testing.

## Methods

This study followed the PRISMA-ScR (Preferred Reporting Items for Systematic Review and Meta-Analyses for Scoping Reviews) guidelines [8]. We reviewed papers on South Korea’s response to COVID-19 to capture the concept of tracing and testing. Papers that addressed the epidemiological investigation process in South Korea from 2020 to 2021 with the number of tests and cases, were included. In addition, a conceptual model was developed based on the concept of tracing and testing. SEIR model was used, and quarantine was included to the model for further understanding the process of tracing and testing. We gathered COVID-19 risk indicators from KDCA (Korea Disease Control and Prevention Agency) press releases issued between June 2020 and February 2022 to validate the accuracy of our developed model’s hypotheses.

### Search strategy

The search terms were combined with terms related to South Korea’s response to COVID-19. A search of studies on databases was performed on 17th October 2021, including Pubmed and Embase. The detail search terms for Pubmed are presented in Supplementary material (Table S1).

### Inclusion and exclusion criteria

In this review, we included studies on South Korea’s response to COVID-19 outbreaks in 2020. Studies that did not include examples of South Korea and did not report the number of confirmed cases and tests were excluded. Additionally, studies not written in English were excluded. Conference abstracts, review paper, letters, editorials, or article comments were excluded. The detail inclusion and exclusion criteria are presented in Supplementary material (Table S2).

### Screening

An author firstly screened each study by title and abstract according to inclusion criteria using reference management software, EndNote 20.2.1 version. After the first screening, another author independently conducted the second screening. The full text of the title and abstract screened studies was reviewed by all authors.

### Data extraction

To clarify the tracing and testing strategies of South Korea, the number of cases and tests were extracted from the reviewed literatures. The process of tracing and testing in each study were extracted to describe and understand the strategic changes of South Korea’s response in 2020.

## Results

### Results of search and screening

The search on databases identified 2,971 studies, and 472 duplicates were removed. In title and abstract screening, 2,200 studies were excluded because they were not about COVID-19 or South Korea. In the full-text screening, 293 studies that did not report the number of cases and tests were excluded. Finally, 6 studies were selected for this review.

### South Korea’s response to COVID-19

South Korea has expanded its testing capabilities to overcome the limitations of contact tracing. In the early stage, South Korea strengthened quarantine for those arriving from abroad and conducted tests on suspected cases who visited areas of COVID-19 spread or had symptoms related to COVID-19. In addition, through contact tracing and testing, efforts were made to locate exposed persons and sources of infection. On February 7, 2020, three cases were confirmed, and investigation revealed an outbreak related to Zumba dance classes [9]. Epidemiological investigators traced 1,687 contacts, 116 of which were confirmed. In addition, eight Zumba instructors were identified as sources of infection [9]. However, as the number of confirmed cases increased, South Korea’s contact tracing capability was exceeded.

The government of South Korea actively responded by tracing groups at risk of infection and testing all individuals in those groups. The first COVID-19 wave in South Korea began in Shincheonji Church (hereafter S. Church) in Daegu [10]. Although the index case of S. Church had symptoms related to COVID-19, she was tested late, and the source of her infection was not identified [10]. As a result of contact tracing of the index case, about 1,000 persons who attended the same worship service were classified as contacts and tested. However, as the number of confirmed cases in S. Church increased, the health authorities decided to test all members and related persons (*n* = 10,220) based on the potential for a large-scale outbreak in S. Church [10]. As a result, 4,137 cases were confirmed [10].

Tracing of groups and large-scale testing of all members continued in South Korea. After a worker in a call center in Seoul was confirmed to have COVID-19 in March 2020, epidemiological investigators determined that the possibility of an outbreak in the call center was high, based on its workplace environment characteristics [11]. All workers in the call center, as well as residents of and visitors to the building, were tested (*n* = 1,143); 96 cases were confirmed [11]. Related to this call center outbreak, a nurse at a long-term care hospital in Bucheon, South Korea, was confirmed to have COVID-19 [12]. 22 hospital workers with the same working hours, and all residents, were classified as contacts. All workers and residents were tested (*n* = 227), and there were no additional cases [12]. In March 2020, three confirmed cases were reported among visitors to a spa facility in Cheonan [13]. The health authority conducted tests on all workers and visitors (*n* = 2,245) to the spa facility and building. As a result, seven confirmed cases were identified [13]. A large-scale testing strategy was implemented after the Itaewon club outbreak in May 2020 [14]. After social distancing was relaxed in South Korea on May 6, 2020, confirmed cases continued to occur at several clubs in Itaewon, Seoul [14]. In response, the Seoul Metropolitan Government and health authorities conducted nationwide large-scale testing by tracing all visitors to the clubs; 41,612 persons were tested, and 96 cases were confirmed [14].

Temporary screening centers were used in South Korea to prevent sporadic infections in the community. Temporary screening centers were operating in the Seoul metropolitan area at the beginning of the third wave, and as the number of confirmed cases increased, these centers were established nationwide. Unlike previous waves, the third wave in South Korea was driven by a small community outbreak with an unknown source of infection [15] and was spread by pre-symptomatic and asymptomatic cases. Therefore, suppression by tracing became difficult, and temporary screening centers were introduced to identify pre-symptomatic and asymptomatic cases in the community.

South Korea’s testing strategies are based on the risk of infection determined by tracing. The epidemiology investigators, who traced COVID-19 outbreaks, defined risk group, and planned how to test persons relating to the risk group. Tracing and testing strategies of reviewed studies are listed in Table 1. Two examples outside of South Korea are included to demonstrate that comparable approaches were sometimes utilized in other countries [16, 17]. Normally, South Korea tested all persons who had increased risk, which is higher risk than background risk.

**Table 1:** Testing strategies and risk of infection

				Risk of infection^a^				
Author (year) [Ref]	Place	Risk group	Background risk^b^	Increased risk^c^	High risk^d^	No. tested	No. cases	Key strategy
Bae S et al. (2020) [9]	Cheonan, Korea	Fitness center	Non-visitors	All visitors and workers in buildings	**Students of the Zumba classes**	1,687	116	Bidirectional tracing, tracing related testing, Quarantine
Kim JY et al. (2021) [10]	Daegu, Korea	Shincheonji church	Non-members	**All members**	Members who attended the worship services as the index case	10,220	4,137	Group tracing, preemptive testing, Quarantine
Park SY et al. (2020) [11]	Seoul, Korea	Call center	Non-visitors	**All visitors, workers, and residents in the building**	Workers in the call center	1,143	97	Group tracing, preemptive testing, Quarantine
Kim T (2020) [12]	Bucheon, Korea	LTCH^e^	Non-visitors	**All residents and staff**	Residents and staff who had close contact with the index case	227	0	Group tracing, preemptive testing, Quarantine
Han T (2020) [13]	Jinju, Korea	Spa facility	Non-visitors	**All visitors and workers in the building**	People who were in the spa facility at the same time as the confirmed cases	2,245	10	Group tracing, preemptive testing, Quarantine
Kang CR et al. (2020) [14]	Seoul, Korea	Itaewon nightclubs	All residents and visitors in Itaewon	**All visitors of nightclubs and people who were in the vicinity of nightclubs for > 30 min**	Unknown	41,612	246	Group tracing, preemptive testing, Quarantine
Telford CT et al. (2020) [16]	Georgia, USA	LTCFs^f^	Non-members	**All residents and staff**	Residents and staff with symptoms	2,022	15	Group tracing, preemptive testing
Cao S et al. (2020) [17]	Wuhan, China	Wuhan	**All residents**	Unknown	Unknown	9,899,828	300	Group tracing, preemptive testing

^a^Bold text indicates testing subject of each reviewed literature

^b^Risk of infection in unexposed individuals

^c^Risk of infection in individuals suspected of exposure

^d^Risk of infection in exposed individuals

^e^*LTCH* Long-term care hospital

^f^*LTCFs* Long-term care facilities

### Contact tracing with tracing-related testing

**Fig. 1:**
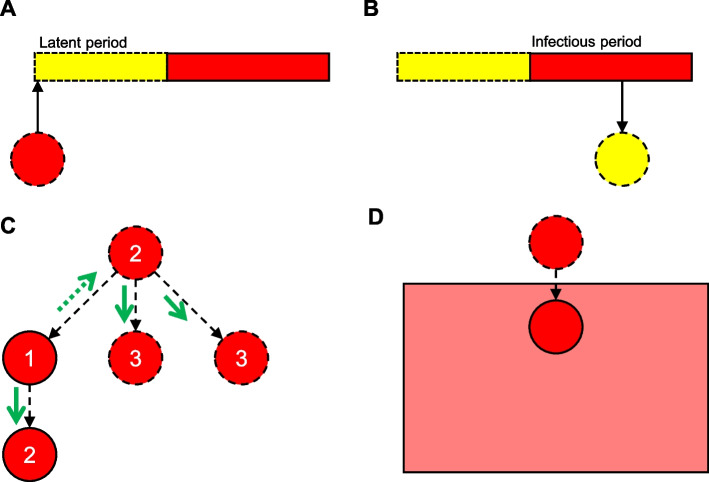
Types of tracing and source of infection. **A** Backward contact tracing. Backward tracing attempts to identify the primary case as the source of infection by finding contacts during the latent period of a confirmed case; dashed red circle, unknown primary case. **B** Forward contact tracing. Forward tracing identifies and quarantines contacts during the infectious period of a confirmed case. Dashed yellow circle, contact of a confirmed case during the infectious period. **C** Forward and backward tracing from an index case (no. 1). Contacts (no. 2) of the index case are identified by forward (solid green arrow) and backward (dashed green arrow) contact tracing. Additional cases (no. 3) are identified by forward tracing of a case (no. 2) identified by backward tracing of the index case. **D** Group tracing. Group tracing refers to the tracing of a group suspected of being a COVID-19 cluster. Red box, potential cluster; dashed red circle above box, unknown primary case; solid red circle, index case of the potential cluster

South Korea’s approach to combining tracing and testing encompassed 3 strategies: contact tracing with tracing-related testing, group tracing with preemptive testing, and testing of untraced individuals. Contact tracing with tracing-related testing can identify persons suspected of having been in close contact with an infected individual, assess their exposure, and quarantine them. Contact tracing is divided into backward and forward tracing. Backward tracing identifies the source of infection, and locates contacts during the latent period of a confirmed case (Fig. 1a) [18–20]. Backward tracing strengthens the effectiveness of the overall response by identifying clusters that were not found by forward tracing, but it is often challenged to find the source of infection because it relies primarily on reporting of index case. Forward tracing identifies contacts exposed to an infector and locates individuals in contact with the infector during the infectious period (Fig. 1b) [18–20]. As forward tracing is a proactive approach, it has a preventive effect due to rapid detection of contacts, but for infectious diseases with high R_0_, it is difficult to lower R_t_ only by forward tracing. In South Korea, bidirectional tracing, *i.e.*, combination of forward and backward tracing, was performed (Fig. 1c) [20]. In addition, traced contacts were tested immediately, and negative contacts were quarantined until their potential infectious period ends. South Korea contained the spread of COVID-19 in the early stage of the pandemic by bidirectional contact tracing and quarantining contacts with tracing-related testing.

### Group tracing with preemptive testing

Group tracing with preemptive testing is defined as tracing a group suspected of outbreak and testing those related to the traced group (Fig. 1d) [21]. A group suspected of outbreak is one in which the risk of infection is greater than the background risk, which is the risk of general population. Digital information such as GPS data, mobile data signals, and credit card usage history was utilized for tracing, which enabled particularly large-scale group tracing [22]. A group with increased risk is defined as a risk group and preemptive testing on individuals in the risk group is conducted. Preemptive testing refers to the screening of all persons in the traced group and is performed irrespective of symptom onset or exposure assessment of the individuals. Quarantining of persons in the traced group is optional, and the optimal timing for testing is before and after quarantine. Preemptive testing was implemented not only in South Korea, but also in other countries to identify asymptomatic cases [16, 17, 23]. Preemptive testing suppressed the spread of COVID-19 in long-term care facilities in the United States [16]. In Wuhan, China, after the lockdown, all citizens were tested (*n* = 9,899,828), and 300 asymptomatic cases were identified [17] (Table 1). As such, group tracing with preemptive testing effectively found presymptomatic and asymptomatic cases in the risk group who were not detected by contact tracing. Group tracing with preemptive testing served as a complementary strategy to contact tracing.

### Testing of untraced individuals

**Fig. 2:**
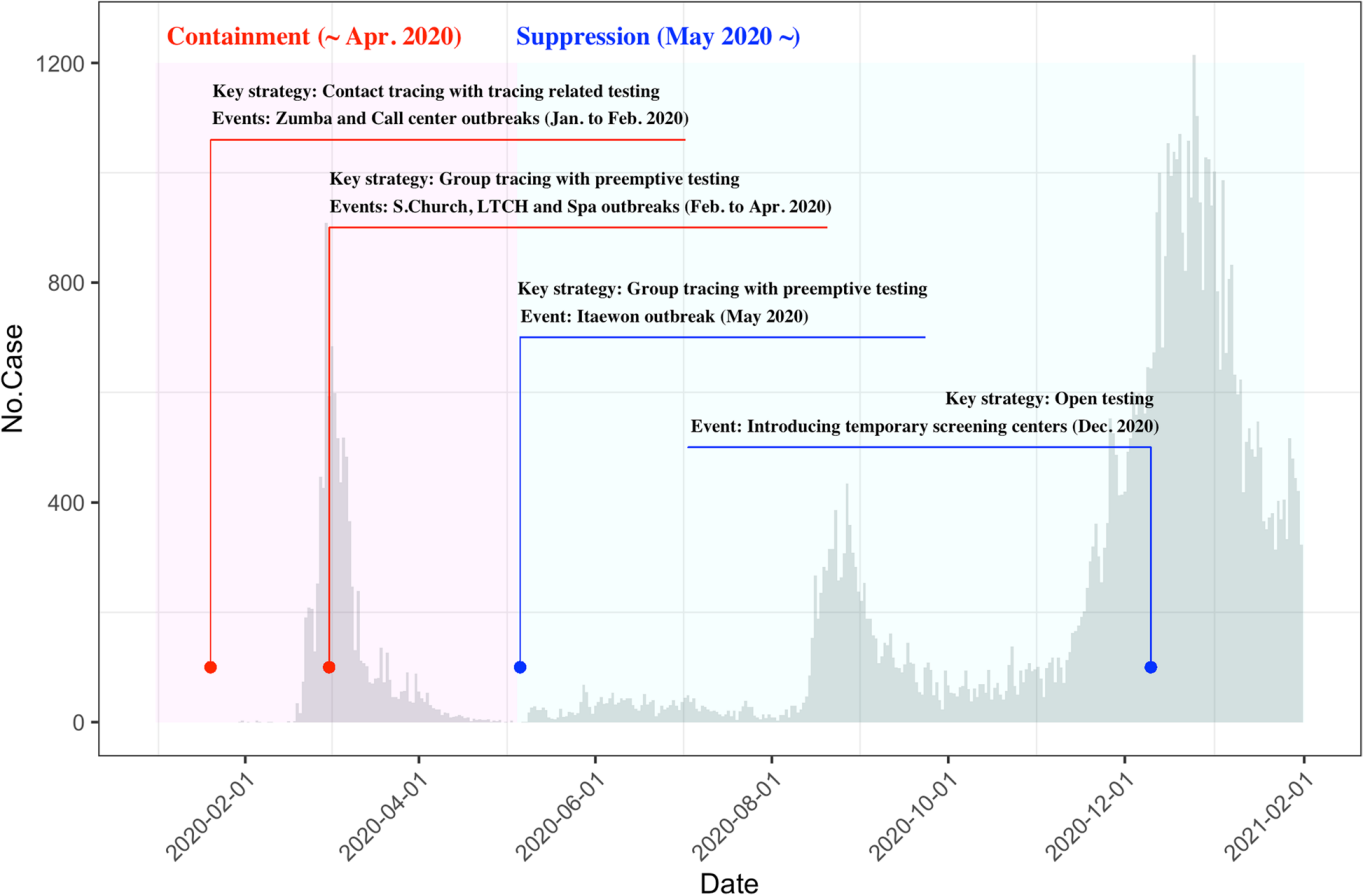
Key strategic changes in South Korea

Untraced individuals are categorized as 2 groups: individuals who meet the criteria for a suspected case (testing on persons who visited areas of COVID-19 spread and/or had symptoms related to COVID-19), and individuals who do not meet the criteria for a suspected case. At the beginning of the pandemic, testing was performed only on untraced individuals who met the criteria for a suspected case (suspected case testing). The index cases of the above-mentioned outbreaks were identified by suspected case testing. As a result, suspected case testing contributed to suppress the transmission in South Korea. Because it is difficult to test pre-symptomatic and asymptomatic cases, South Korea allowed the testing of individuals who did not meet the criteria for a suspected case. This strategy (testing anyone who wishes to be tested regardless of epidemiological association) is defined as open testing and enables detection of pre-symptomatic and asymptomatic cases. As open testing was implemented by the temporary screening centers nationwide, the number of tests increased and the transmission of COVID-19 decreased [24, 25]. The key strategic changes in South Korea were shown in Fig. 2.

### Conceptual model based on South Korea’s experience

#### SEQIR model

**Fig. 3:**
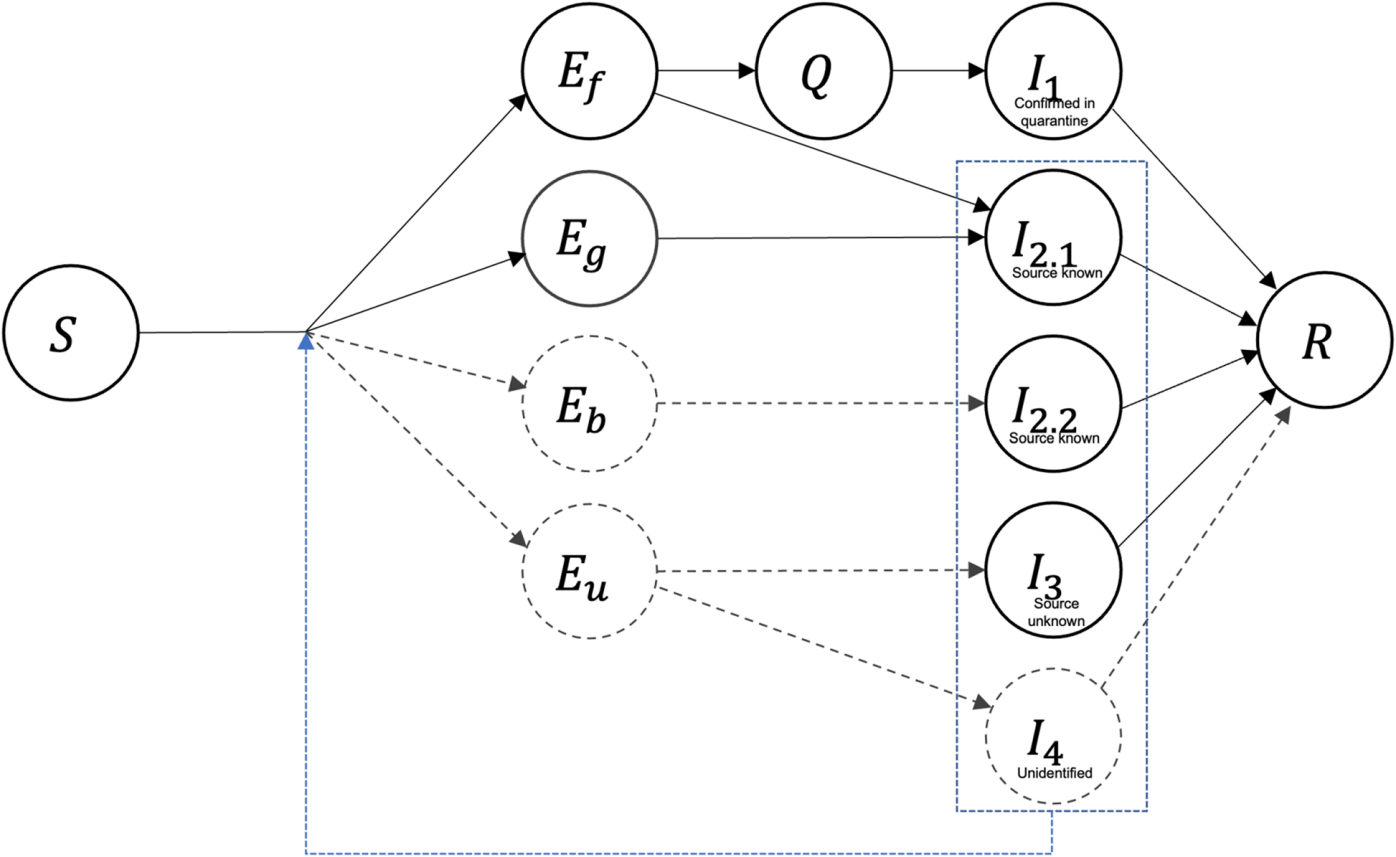
SEQIR model of tracing and testing. Exposure compartments are classified as E_f_, E_b_, E_g_, and E_u_ based on the type of contact tracing (subscript; forward tracing (f), backward tracing (b), group tracing (g), untraced (u)). Infection compartments are classified as I_1_–I_4_, according to tracing, testing and quarantine. I_1_ is confirmed during quarantine by forward contact tracing with tracing-related testing, and has no additional transmission due to timely quarantine. I_2_ is a confirmed case not under quarantine with a known source of infection. Among the contacts identified by forward contact tracing, a case confirmed without quarantine takes the first path of I_2_ (E_f_ → I_2.1_). The second path of I_2_ is taken by a case confirmed by tracing of a group suspected to be a COVID-19 cluster (E_g_ → I_2.1_). For this case, the cluster is designated as a source of infection. The third path of I_2_ corresponds to the source of infection being identified by backward contact tracing after confirming a case (E_b_ → I_2.2_). I_3_ is a confirmed case with an unknown source of infection, and I_4_ is an unidentified case that has not been traced, quarantined, or tested. Dotted line, unobserved state; solid line, observed state. Case types within a dotted box may transmit infection to others, as indicated by the feedback arrow

We developed a model based on tracing, testing, and quarantine in South Korea (Fig. 2). The proposed model has five compartments: S, E, Q, I, and R. S denotes susceptible individuals without immunity to COVID-19 by vaccination or natural infection. People who have been in contact with an infected person but are not yet infectious move from S to E. The movement from S to E is determined by parameters such as transmission rate, infection period, and contact rate between people. E refers to persons exposed to COVID-19 subject to contact and group tracing. The tracing concepts described above are presented as subscripts of each E compartment in Fig. 3. The movement from E to I is affected by latent period. Cases confirmed by testing are in the infected group, and are classified into four types according to the process of tracing, testing, and quarantine. As shown in Fig. 3, the type of I affects key parameters such as infection period and contact rate. Infected group moved to recovery compartment by recovering rate. Unknown parameters such as quarantined proportion, proportion of each E compartment, and proportion of unidentified cases (I_4_) can be calibrated or estimated using real data.

#### Case types by proposed model

I_1_ was defined as cases confirmed in quarantine (Fig. 3). Some contacts were identified before their infectious period by forward tracing and quarantined to prevent further spread of COVID-19. I_2_ was defined as a non-quarantined cases with a known source of infection. I_2_ was divided into I_2.1_ and I_2.2_ depending on whether the source was known at the time of confirmation. I_2.1_ was identified by forward and group tracing and confirmed positive for COVID-19 before quarantine. I_2.2_ was not traced, so the source was unknown at the time of confirmation but later identified via backward tracing. Since backward tracing is conducted after confirmation, the number of I_2.2_ among new confirmed cases reported on any given day is unknown, and I_2.2_ can be distinguished from I_3_ after a few days.

I_3_ was a case not detected by tracing, and for which the source was not traced after confirmation. Persons who participated in suspected case testing or open testing did not have an epidemiological linkage. The source of infection was unknown at the time of confirmation. Also, backward tracing failed due to recall bias and the high rate of asymptomatic cases [10, 18, 19].

Cases not traced and tested were in the unidentified group (I_4_), and were not included among the confirmed cases. The types of cases confirmed by tracing, testing and quarantine are shown in Table 2; these types were applied to the reviewed papers (Table 3). I_1_ applied to two cases from the spa facility outbreak and 108 from the fitness center outbreak. All confirmed cases in the S. Church, call center, and Itaewon club outbreaks who underwent group tracing and preemptive testing were classified as I_2_. Because detailed classification is hampered by the lack of information on the date of confirmation and quarantine, the index cases of these outbreaks were also classified as I_2_. In addition, five cases in the spa facility and three in the fitness center were classified as I_2_. Finally, three index cases in the spa facility outbreak and eight Zumba instructors identified as sources of infection in the fitness center outbreak were classified as I_3_.

**Table 2:** Tracing, testing, and quarantine strategy to identify types of COVID-19 cases in South Korea

Category	Tracing	Testing	Quarantine	Case type^a^	Details of case type
1	Forward tracing	Tracing-related testing	Quarantine	${I}_{1}$	Confirmed in quarantine
2	Forward tracing	Tracing-related testing	Non-quarantine	${I}_{2.1}$	Source known
3	Backward tracing	Tracing-related testing	Non-quarantine	${I}_{2.1}$	
4	Group tracing	Preemptive testing	Non-quarantine	${I}_{2.2}$	
5	Untraced	Suspected case testing	Non-quarantine	${I}_{3}$	Source unknown
6	Untraced	Open testing	Non-quarantine	${I}_{3}$	

^a^See text for detailed definitions of case types

**Table 3:** Numbers of I_1_, I_2_, and I_3_ cases

Author (year) [Ref]	${I}_{1}$	${I}_{2}$	${I}_{3}$	cases
Bae S et al. (2020) [9]	108	3	8	119
Kim JY et al. (2021) [10]	0	4,137	0	4,137
Park SY et al. (2020) [11]	0	97	0	97
Han T (2020) [13]	2	5	3	10
Kang CR et al. (2020) [14]	0	246	0	246

#### Novel indicators based on the SEQIR model

**Fig. 4:**
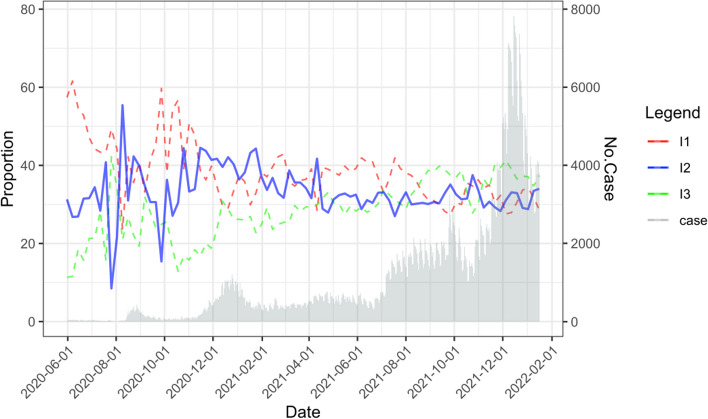
Time series trends of the case types in South Korea. The rate of I_3_ decrease (green dashed line) was moderated by increasing I_2_ (blue solid line) when I_1_ (red dashed line) was lowered during the spread of COVID-19 (third wave, November 2020 to January 2021; fourth wave, July to October 2021)

The proportions of case types can be used as indicators for tracing and testing. Because the case types are defined by tracing and testing, the performance thereof can be assessed based on the proportion of each case type. First, the overall effectiveness of tracing and testing can be determined based on the proportion of I_1_ and I_2_ among confirmed cases (I_1_ + I_2_ + I_3_). This measure is termed as “traced proportion”. In South Korea, traced proportion remained above 60% due to extensive tracing and large-scale testing (Fig. 4). In the Fig. 4, I_3_ (the green dotted line) rarely rise over 40%, and this means that the traced proportion (I_1_ + I_2_ = 100%-I_3_) is maintained generally above 60% in this period. In addition, the relative proportions of case types can indicate the tracing and testing capabilities that need to be enhanced.

**Fig. 5:**
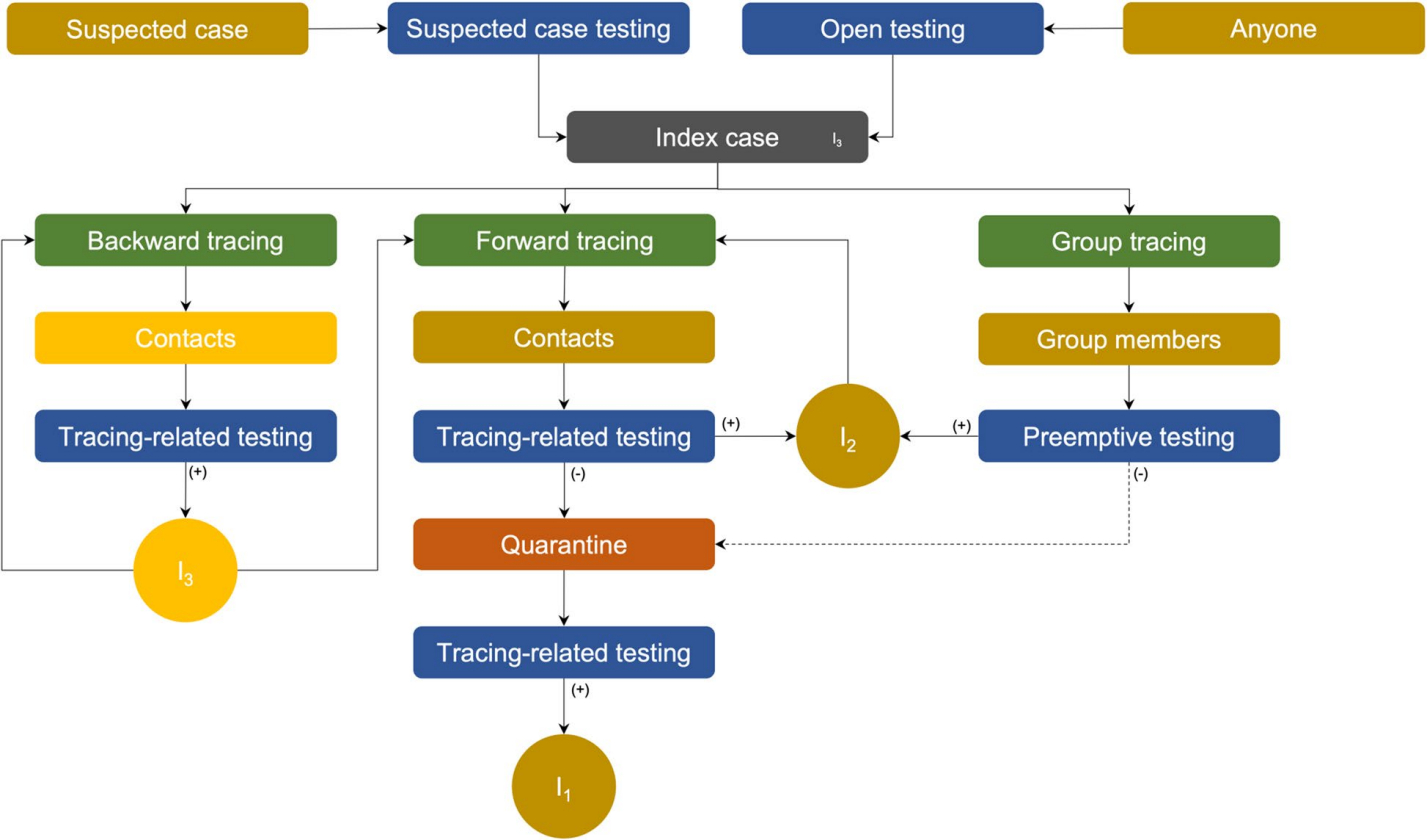
Tracing and testing algorithm. The index case was identified by suspected case testing or open testing. Backward and forward tracing were performed to identify contacts of the index case. Cases confirmed by backward tracing had an unknown source (I_3_), so backward tracing was repeatedly performed until no additional cases were found. Contacts identified by forward tracing were confirmed before quarantine, and forward tracing was repeated until no additional cases were found. Cases confirmed in quarantine are the end point of the algorithm, indicating no further spread of infection. Cases identified by group tracing and preemptive testing had a known source, and required further forward tracing. Cases in quarantine can be identified by group tracing or preemptive testing depending on the guidelines applied. For example, all members of Shincheonji Church were instructed to self-quarantine by the health authority before undergoing preemptive testing

I_1_ is the endpoint of tracing and testing process (Fig. 5). A strategy conducting contact tracing and quarantine with testing before and after quarantine to increase the proportion of I_1_ is an effective strategy to prevent the spread of COVID-19 transmission early [6, 26, 27]. Additionally, with sufficient tracing and testing, this strategy can effectively prevent I_2_ and I_3_, and reduce contacts by minimizing the time confirmed cases spend with others. Thus, I_1_ is the first metric to monitor.

I_2_ includes cases of delayed forward and group tracing. If tracing is delayed, additional forward tracing is required to find secondary infections. Therefore, the continuous occurrence of I_2_ may lead to an iterative forward-tracing. To break this, acceleration of forward tracing, or wider quarantine of individuals belonging to traced groups are needed.

I_3_ refers to untraced cases. The proportion of I_3_ may be increased by an accumulation of undetected cases in the community, and by high proportions of pre-symptomatic and asymptomatic cases. An increase in I_3_ needs to be prevented because it may lead to large-scale outbreaks by promoting silent transmission. Strengthening of rapid contact tracing and implementing group tracing and preemptive testing can prevent an increase in I_3_. In addition, large-scale open testing can reduce the proportion of I_3_ by identifying pre-symptomatic and asymptomatic cases in the community. Furthermore, a large proportion of I_3_ implies that the tracing capability is poor compared to testing. Therefore, the proportion of I_3_ can be an alternative indicator to the traced proportion when a country’s tracing capability is insufficient.

## Discussion

In this study, we evaluated tracing and testing in South Korea by analyzing the response to COVID-19 outbreaks. In South Korea, forward and backward tracing were implemented. In addition, group tracing combined with preemptive testing and open testing were conducted to overcome the limitations of conventional contact tracing. We proposed the SEQIR model to explore the properties of case types according to tracing and testing strategies. In the model, the confirmed cases were classified into I_1_–I_4_, the proportions of which can be used as tracing and testing performance indicators.

In South Korea, simultaneous forward and backward tracing was an effective countermeasure for COVID-19—backward tracing can identify cases missed by forward tracing (Fig. 1c). This is consistent with prior studies that bidirectional tracing allows the detection of hidden transmission paths [18, 20]. Moreover, bidirectional tracing was superior for controlling the spread of COVID-19 compared with forward tracing alone [18, 20]. The proportion of I_1_ in South Korea remained almost above 90% from March 2020 to April 2020, which was not included in Fig. 4. This shows that South Korea proactively identified almost all cases by bidirectional contact tracing in the early stage of pandemic as shown in Fig. 2.

The case types in this study were consistent with South Korea’s risk assessment indicators. The proportion of I_1_ is identical to the timely quarantined proportion (TQP) proposed previously, and can be used to assess the effects of epidemiological investigation, testing, and quarantine [28]. It is necessary to monitor trends in I_1_ to prevent the spread of COVID-19.

Maintaining a high proportion of I_1_ is a challenge for many countries. Furthermore, maintaining a high proportion of I_1_ during the period of delta variant predominance was problematic because of its high transmission rate and ability to escape the immune system. The emergence of a new variant can increase the proportion of untraced cases (I_3_). An unlinked case is a confirmed case with no link to the infector [29, 30]. A confirmed case discovered by group tracing and preemptive testing can be classified as an unlinked case, but not as an untraced case, and remains controllable. Reducing the untraced proportion is another major challenge, but can be achieved by increasing the proportion of I_2_.

Proactive and fast tracing is necessary to increase I_2_. However, as mentioned above, the emergence of new variants can hamper the tracing of individuals suspected of having close contact with confirmed cases. In this case, group tracing and preemptive testing is a feasible alternative strategy to identify super-spreaders and reduce cluster size. During the period of delta variant predominance (after 2021 July) in South Korea, I_1_ decreased, but I_3_ remained < 40% (Fig. 4). According to the proposed model, this was achieved by group tracing and preemptive testing.

I_4_ is one of the major concerns to control the COVID-19 transmission. There are several studies on the estimates of I_4_. Lee et al. estimated the proportion of undetected case of COVID-19 in South Korea as 5.8% (5,200/89,244) to 64% (139,900/218,744) using data as of 2nd February 2021, and a probabilistic model they developed [31]. A modeling study conducted by Huo et al. estimated the proportion of asymptomatic and undetected case in Wuhan, China as 22.4% (14,448/64,454) [32]. A systematic review which analyzed 79 studies, estimated the proportion of asymptomatic case as 20% (95% C.I 17%­25%) [33]. Additionally, these studies revealed that I_4_ has transmissibility [31, 32], and this was shown in the reviewed studies. The sources of infection of index cases in S. Church, call center, spa facility, and Itaewon nightclubs outbreaks were not identified [10, 11, 13, 14]. Therefore, it is necessary to consider in the response planning not only the identified cases, but also the unidentified cases.

Unlike previous works, this study described the tracing and testing process in detail. In addition, the proposed model may be useful for other countries. However, we did not address the social distancing and vaccination policies that were instrumental for flattening the COVID-19 curve. In addition, statistical analysis of empirical data was not performed because the current study focused on conceptual analysis of South Korea’s COVID-19 tracing and testing strategies. Lastly, as this was not a systematic review, it did not include all articles that analyzed South Korea’s response to COVID-19.

## Conclusion

South Korea responded to COVID-19 by expanding its testing capabilities. Group tracing with preemptive testing complemented traditional contact tracing. Open testing enabled detection of pre-symptomatic and asymptomatic cases. Finally, we found four case types, and the proportions of case types among confirmed cases could be used as indicators to of the effectiveness of tracing and testing; maintaining a high traced proportion is vital for the suppression of COVID-19 transmission.

### Supplementary Information

**Supplementary Material 1.**

## Data Availability

The datasets used and/or analyzed during the current study are available from the corresponding author on reasonable request.
