# Supplementary material for: New framework to assess tracing and testing based on South Korea’s response to COVID-19
Source: BMC Infect Dis. 2024 May 3;24:469. doi: 10.1186/s12879-024-09363-4 (PMC11067276; doi:10.1186/s12879-024-09363-4)
Supplement: Supplementary file 1 — Supplementary Material 1. [file 12879_2024_9363_MOESM1_ESM.docx]

**Supplementary material**

Table S1. The search terms used in this study

| # | Search terms |
| --- | --- |
| #1 | COVID-19[MeSH] |
| #2 | ("Non pharmaceutical intervention"[ALL] OR NPI[ALL] OR "Control measure"[ALL] OR "Preventive policy"[ALL] OR "Public health intervention"[ALL]) |
| #3 | (Testing[ALL] OR "COVID-19 testing"[ALL] OR "RT-PCR"[ALL] OR PCR[ALL] OR "Aggressive testing"[ALL] OR "Screening center"[ALL] OR "Drive through"[ALL] OR "Walk through"[ALL] OR "Laboratory diagnosis"[ALL]) |
| #4 | ("Contact tracing"[ALL] OR Tracking[ALL] OR "Epidemiological invesigation"[ALL]) |
| #5 | Isolation[ALL] |
| #6 | (Quarantine[ALL] OR Quarantine[MESH] OR "Border closure"[ALL] OR "Travel ban"[ALL] OR "Travel restriction"[ALL]) |
| #7 | ("Social distancing"[ALL] OR "Social distance"[ALL] OR "Physical distancing"[ALL] OR "Physical distance"[ALL] OR "School closure"[ALL] OR "Work closure"[ALL] OR "gathering"[ALL]) |
| #8 | (effect* OR Impact OR influen*) |
| #9 | (statist* OR analy* OR model* OR simul*) |
| #10 | (reduc* AND transmission) |
|  | #1 AND (#2 OR #3 OR #4 OR #5 OR #6 OR #7) AND ((#8 OR #9) AND #10) |

Table S2. Inclusion and Exclusion Criteria

| Criteria | Inclusion | Exclusion |
| --- | --- | --- |
| Population | COVID-19 cases and their contacts | Other diseases |
| Concept | Response to COVID-19 outbreak with the number of confirmed cases and tests | Did not report the number of confirmed cases and tests |
| Context | South Korea | Did not include South Korea |
| Others | Published in scientific journals,  Written in English | Conference abstracts, Review paper,  Letters, Editorials, Article comments |
